# Exploring the Conditions and Strategies That Lead Nurses to Advocate for Elderly Cancer Patients in the Perioperative Intensive Care Unit: A Qualitative Study

**DOI:** 10.3390/healthcare13222848

**Published:** 2025-11-10

**Authors:** Sara Morais Pires, Idalina Gomes

**Affiliations:** Nursing Research, Innovation and Development Centre of Lisbon (CIDNUR), Nursing School of Lisbon (ESEL), 1600-096 Lisbon, Portugal

**Keywords:** patient advocacy, grounded theory, geriatric oncology, perioperative care, intensive care unit

## Abstract

**Background/Objectives:** Elderly cancer patients in perioperative intensive care units represent a highly vulnerable population due to complex medical needs and physiological challenges. In these high-pressure settings, nurses play a crucial role as patient advocates to ensure well-being and person-centered care. However, there is limited understanding of how nurses operationalize this advocacy role for this specific patient group. This study aimed to explore the conditions and strategies guiding nurses in advocating for elderly cancer patients in perioperative ICU contexts. **Methods:** Using a Grounded Theory approach, six nurses with direct experience in caring for elderly cancer patients in an oncology institution were recruited. Data were collected via in-depth semi-structured interviews, participant observation, and field notes. Analysis followed the constant comparative method and was supported by MAXQDA software to systematically identify codes, dimensions, and categories, highlighting essential elements of nursing advocacy. **Results:** Advocacy emerged as a central nursing function, serving both practical and ethical imperatives. Conditions activating advocacy included patients’ complex vulnerability, threatening dignity, mitigated by the family’s anchoring role. Institutional barriers, such as professional devaluation and staffing shortages, often hindered advocacy, resulting in moments of “failure to advocate.” Strategies formed a dynamic process: beginning with in-depth knowledge, progressing through communication mediation, and culminating in direct action. **Conclusions:** This study contributes to the development of a data-driven theory that deepens the understanding of nursing advocacy in perioperative intensive care for elderly cancer patients, offering valuable implications for practice, education, and policy development, ultimately supporting optimized care delivery and improved patient outcomes.

## 1. Introduction

Population ageing and the growing incidence of cancer make elderly patients a particularly vulnerable group, especially when undergoing highly complex surgical procedures and admission to intensive care units (ICUs) [1].

The elderly have a high vulnerability profile, with deficits concentrated in mobility, activities of daily living, and cognitive status. Factors such as advanced age (≥85 years), low educational attainment, and depressed emotional state have been identified as significant predictors of greater functional impairment, with a large percentage requiring full compensation for severe self-care deficits [2,3,4]. Additionally, these studies have shown that functionality can be dynamic and time-dependent. Although there may be significant improvements in the initial rehabilitation period, functionality tends to decline after prolonged hospitalization. These findings underscore that, given the complexity and rapid changes in functional and cognitive status, the elderly, particularly those with cancer in the highly critical environment of the Perioperative Intensive Care Unit, constitute a high-risk group. This population faces a state of Complex Vulnerability, stemming not only from polypharmacy and the direct effects of chemotherapy and surgery, but also from the overall emotional burden and the threat to personal dignity imposed by diagnosis and treatment regimens. Consequently, continuous monitoring and active advocacy intervention by nurses are fundamentally justified to mitigate these profound effects, ensure individualized care, and maintain the patient’s quality of life [5,6].

Advocacy in nursing, understood as the defense of the rights, autonomy and safety of the patient, emerges as an essential element of professional practice, especially in scenarios of high clinical vulnerability, such as oncological surgery in the elderly [6,7]. The evidence is compelling in defining safeguarding, effective communication, empowerment and support for ethical decision-making as central attributes of advocacy, these aspects being critical to ensuring the dignity and active participation of older people in the therapeutic process [6,7,8].

The conditions that enhance advocacy include nurses’ professional autonomy, continuing education, interprofessional collaboration, and spiritual and communication skills, which enable them to respond to the complex needs of older adults in the perioperative setting [9,10]. A systematic review focusing on the role of oncology nurses in the decision-making process of elderly people with cancer identified three main functions, namely geriatric assessments, the provision of adequate information, and advocacy [11].

In this context, factors such as frailty, multimorbidity, cognitive limitations, and functional dependence increase the risk of postoperative complications and the need for person-centred care [12]. This vulnerability is not restricted to the clinical dimension, but also extends to the ethical and relational fields, requiring healthcare professionals, especially nurses, to act with sensitivity, specialised knowledge and active defence of the rights of older people with cancer [13].

In the particular case of elderly people with cancer, advocacy takes on even greater relevance, given that clinical decisions often involve ethical dilemmas, invasive options and significant risks to quality of life. The role of the nurse as mediator, educator and advocate is therefore crucial to ensure clear information, respect for patient preferences and the integration of the family into the care process [14,15].

In this highly complex and interdependent scenario, nurses play a crucial role and are required to take an active role in advocating for patients and their needs. However, barriers such as work overload, lack of time, cultural or religious conflicts, and ineffective communication may limit nurses’ ability to intervene [16,17].

However, the success of this role not only impacts the quality of care, but also directly influences the health and professional satisfaction of the nurse themselves, with failure being a source of emotional and psychological consequences that compromise care. This web of interdependence requires a thorough understanding of the dynamics of care and the role of nurses in optimising health outcomes in the system [16,17,18].

A recent systematic review identified 11 randomised clinical trials on perioperative support interventions in elderly cancer patients undergoing surgery. The most common strategies included exercise programmes, geriatric assessment, nutritional optimisation and patient empowerment, but there was considerable heterogeneity in the methods and outcomes evaluated. Despite some reported benefits, the studies faced limitations such as high dropout rates and difficulties in implementation [10]. These findings reinforce the need for more robust research focused on the specificities of the geriatric cancer population.

Given this lack of evidence on effective perioperative support, and given the ethical relevance and direct impact of advocacy on nurses’ professional well-being, it is crucial to explore in depth the conditions and strategies that enable nurses to effectively advocate for older people with cancer in perioperative and intensive care settings. This understanding aims not only to reinforce ethically sustained clinical practices, but also to inform institutional policies and training programmes that ensure the necessary training and support for professionals to perform this role.

## 2. Materials and Methods

### 2.1. Study Design

Qualitative research, based on the constructivist-interpretivist paradigm [19,20], views reality as multiple and subjective, valuing the meanings attributed by participants [21] and subjectivity as a legitimate source of data [22]. Focused on depth rather than statistical generalization [23] it uses naturalistic methods and inductive, interpretative analysis to construct meanings emerging from the field [24,25].

This study employed Grounded Theory as the methodological framework, enabling an in-depth understanding of nurses’ experiences and perceptions regarding advocacy in complex oncology and perioperative care settings. Grounded Theory was chosen for its capacity to generate theory directly from qualitative data while allowing flexibility to explore emerging themes.

Grounded Theory (GT), initially developed by Glaser and Strauss (1967), is an inductive and iterative qualitative methodology that aims to generate theory from empirical data, based on symbolic interactionism and the interpretive paradigm [25]. It is based on the coding and constant comparison of data, categories and properties [25], presenting different methodological strands, from Glaser’s classical approach (1992) to the systematised version of Strauss and Corbin (2015) and the constructivist perspective of Charmaz (2006) [24,25]. It is a method particularly suited to exploring complex social phenomena, and the adoption of the Straussian strand in this study is justified by its more structured and guiding nature.

### 2.2. Researcher Positioning

Given the constructivist stance adopted in this study, it is important to acknowledge the position of the researcher, as the theorization emerged through co-construction between the researcher and the participants. The principal investigator is a nurse specialist in medical–surgical and perioperative nursing, with 18 years of professional experience in pre-, peri- and postoperative settings. Currently, she serves as an invited teacher and is pursuing doctoral studies focused on advocating for older adults with oncological disease in the perioperative context. Her extensive clinical background and academic trajectory provided her with theoretical sensitivity and a deep understanding of the phenomenon under investigation, which were essential in the co-construction of the proposed theory.

### 2.3. Recruitment

Participants were recruited in Portugal, a country located in southwestern Europe with an estimated population of 10 million. The Portuguese healthcare system is mixed, comprising public and private providers integrated into the National Health Service.

For this study, recruitment took place at an international institution for cancer care and research, anonymized as H1. The study was conducted in the Postoperative Intensive Care Unit (ICU) of this internationally recognized oncology institution, a setting specializing in the perioperative management of complex cancer patients. The ICU maintains a specialized capacity of 8 beds and operates with a strict nurse-to-patient ratio of 1:2 (one nurse per two patients). This staffing model is essential given the high acuity and inherent complexity of the patient population, which necessitates frequent advanced monitoring and procedural care. The workload is further characterized by the intense ethical and clinical pressure associated with life-threatening oncology diagnoses. Crucially, the institutional culture actively fosters interprofessional collaboration and open ethical dialogue between nursing and medical teams, providing a foundation of strong ethical and organizational support that facilitates the nurse’s role as a patient advocate.

Initially, an intentional sampling strategy was employed, and subsequently, the process followed an iterative approach guided by theoretical sampling. Before data collection, the principal investigator presented the study at H1, where initial contact with participants was established, observation was conducted, and any questions regarding the research were answered.

The inclusion criteria covered perioperative oncology nurses with more than 6 years of professional experience in nursing care for people undergoing cancer surgery. Nurses who were on sick leave or absent from the workplace at the time of data collection were excluded from participation.

### 2.4. Data Collection

Guided by a symbolic interactionist perspective, this study assumes that reality, social processes, and professional practices are co-constructed through interaction, communication, and shared meaning. Data were collected using a combination of non-participant observation, in-depth interviews, and document analysis, in line with grounded theory methodology.

Observations were carried out in a private oncology institution (H1), focusing on the perioperative care of older adults with oncological disease. Field notes were recorded in a researcher’s notebook, later digitized and analysed, allowing the identification of environmental characteristics, staff actions, and patterns of interaction that shape care practices.

In-depth interviews were conducted with perioperative oncology nurses recognized as experts due to their extensive experience with older patients. Interviews lasted approximately 50–60 min, were audio-recorded, and transcribed verbatim. Most sessions took place in a private setting within the institution, with alternative locations arranged when confidentiality required. Interviews were analysed iteratively as data were collected, enabling refinement of emerging categories and triangulation of findings.

Additionally, institutional and regulatory documents were reviewed to provide contextual understanding of the organizational, social, and procedural environment. This included hospital protocols, perioperative care guidelines, national health regulations, and policies related to patient safety, psychosocial support, and care for older adults undergoing oncological surgery.

### 2.5. Data Analysis

Data analysis began concurrently with data collection, following the principles of grounded theory. Open coding was initially applied to identify concepts, actions, and interactions relevant to the perioperative care of older adults with oncological disease. This was followed by axial coding, which enabled the exploration of relationships between categories and the conditions and strategies present in the context that support nurses in advocating for their patients. Throughout the process, memo writing was used to record reflections, emerging interpretations, analytical questions, and guidance for further data collection.

**Figure 1:**
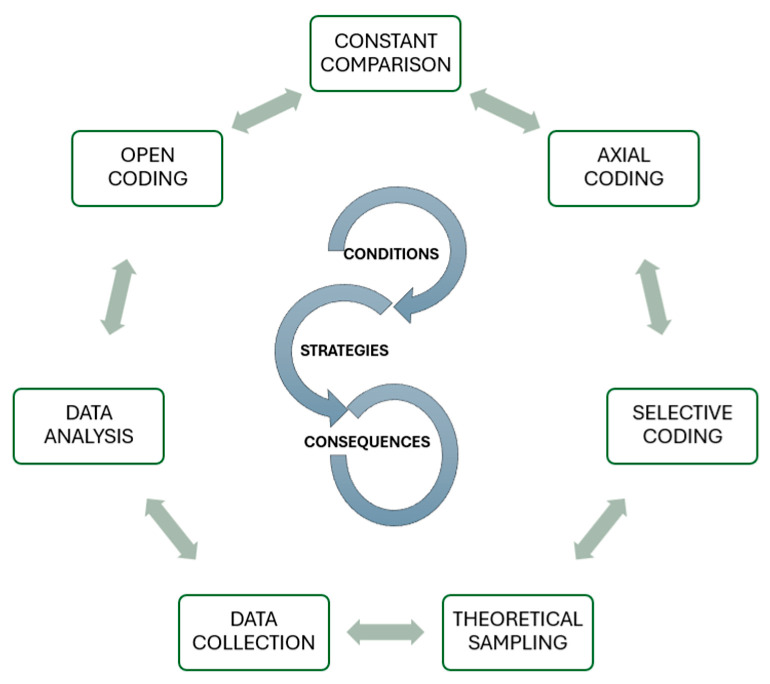
Summary diagram of the research process.

The analytical process was developed in sequential but interconnected phases, beginning with Open Coding. This initial phase aimed to break down the data to identify relevant concepts, actions, and interactions in the perioperative care of elderly people with cancer. Line-by-line coding was applied to the interview transcripts and incident-by-incident coding to the observation notes, generating descriptive codes and in vivo codes. This was followed by Axial Coding, where the emerging categories were regrouped and interrelated (Figure 1.). This stage explored the links between the conditions and strategies identified in the context that support or hinder nurses’ advocacy for patients, using the Coding Paradigm (Conditions, Central Phenomenon and Actions/Strategies) to increase analytical density. The management and coding of all material was performed with the aid of MAXQDA qualitative analysis software.

Throughout the process, Memo Writing was a crucial tool for recording methodological reflections, emerging interpretations, and analytical questions, guiding subsequent theoretical sampling. Coding procedures were adjusted to the type of data (including document-based coding for institutional materials), and codes with greater analytical relevance were refined to increase their abstraction and conceptual clarity. The criterion for ceasing data collection and analysis was theoretical saturation, ensuring the robustness of the model. The Symbolic Interactionism perspective guided the interpretation of the results, focusing on how nurses co-construct meanings and how these influence their advocacy practices.

The sample of six participants was determined by theoretical rather than statistical criteria, focusing on conceptual depth. Theoretical saturation was explicitly confirmed in the sixth interview. This point was established when no new categories, properties, or conceptual relationships emerged, and when the open and axial coding process of the last two interviews only resulted in the consolidation and validation of existing theoretical categories (e.g., Conditions, Strategies, and Advocacy Outcomes), attesting to the stability of the emerging theory. This rigour ensures methodological soundness, despite the small sample size, which reflects the specificity of the intensive cancer care context.

### 2.6. Rigor

The methodological rigour of the study was established through the strict application of the criteria of reliability, transferability, credibility and confirmability, which are essential in Grounded Theory (GT). The approach was conducted in accordance with the guidelines of prominent authors of the methodology, aiming at maximum fidelity to the data.

The credibility of the results, which ensures that the theory accurately reflects the participants’ experience, was ensured by triangulating sources—cross-referencing data obtained from in-depth interviews, non-participant observation, and document analysis. The process of iterative analysis and constant comparison between data incidents and categories allowed the theory to remain anchored in the empirical basis [25]. Fidelity to the participants’ language was reinforced by maintaining in vivo codes throughout Open Coding.

Reflexivity was a central component in maintaining rigour, particularly due to the principal investigator’s insider status in the field of perioperative nursing. To mitigate potential interpretative bias arising from her theoretical sensitivity and clinical experience, Memo Writing was used continuously. These memos served as a reflective diary, documenting the researcher’s preconceptions, analytical decisions, and conceptual evolution to maintain fidelity to the data. Additionally, the process of Formal Analysis and Validation by the second author, a neutral researcher experienced in qualitative research, ensured a second coding and validation of the relationships between categories, strengthening the confirmability and objectivity of the findings.

With regard to analogy, a dense description of the context of the oncology institution and the profile of the participants was provided. This detail allows other researchers and professionals to assess the potential applicability of the theory to similar clinical settings, a fundamental requirement [19]. Theoretical sampling was intentional, recruiting contexts and participants that ensured maximum variation in categories.

Confirmability, which ensures the neutrality of the findings, was guaranteed by the creation of a complete audit trail. This trail was documented through Memo Writing, a crucial technique for recording analytical decisions, the researcher’s reflections, and the progression of the theory, as recommended by Strauss & Corbin [25].

### 2.7. Ethical Considerations

This study was conducted in strict accordance with the ethical principles applicable to research involving human subjects, respecting the rights and well-being of participants. The study protocol was submitted to and approved by the Research Ethics Committee of the partner institution (H1).

To ensure the protection of participants and ethical rigour, strict procedures for informed consent, confidentiality and risk minimisation were implemented. All nurses participating in the interviews were thoroughly informed about the objectives, the method and their right to withdraw from the study at any time. Free and informed consent was obtained in writing before any data collection.

Voluntary participation was guaranteed, with absolute confidentiality being a central pillar. All audio data from the interviews and field notes were immediately coded and anonymised, with pseudonyms assigned to participants and the institution (H1) in all transcripts and publications. Interviews were conducted in private and reserved locations to ensure privacy. In non-participant observation, field notes were recorded in such a way as to avoid identifying any patient or professional involved in care, focusing only on patterns of interaction and advocacy practices.

Data management and storage were carried out in accordance with the General Data Protection Regulation (GDPR). Digital data (audio recordings and transcripts) were stored on secure, password-protected platforms with access restricted to the principal investigators. All measures implemented were aimed at mitigating the risk of emotional overload or exposure of nurses when discussing sensitive topics related to ethical dilemmas and practices in highly critical contexts.

## 3. Results

To frame the qualitative analysis, we first present the sociodemographic and professional characteristics of the participating nurses. The data collected from experts in post-operative oncology care allows us to understand the profile of the interviewees and contextualise their perspectives on nursing advocacy for elderly people with cancer. The description of variables related to age, gender, professional experience, length of practice in an oncology context, and specialised training is an essential element in ensuring methodological transparency and supporting the interpretation of subsequent results (Table 1).

The presentation of results follows the procedural and inductive logic of GT, where data analysis and conceptualization are iterative and interconnected processes. Table 2 summarizes the evolution of analytical coding, demonstrating the systematic transition from descriptive codes (Open Coding) to conceptual categories (Axial Coding) and, finally, to the Central Categories (Selective Coding) that structure the emerging intermediate procedural model. This procedure is essential to ensure methodological transparency and anchor the theoretical product in empirical data.

### 3.1. Conditions

The analysis of the codes extracted using MAXQDA from the data obtained from the interviews revealed a set of key conditions for the exercise of nursing advocacy. The conditions for advocacy emerge as a fundamental pillar of this phenomenon, grouping together the factors that influence or enable this practice and that trigger nurses to take on an active role in advocacy.

The Axial Coding analysis revealed a set of key conditions that trigger and modulate the nurse’s response when exercising advocacy.

➢ Complex Vulnerability

The Complex Vulnerability category constitutes the primordial activating factor for advocacy, summarizing the multidimensional fragility of the elderly oncology patient in the perioperative care environment. This vulnerability strictly transcends the clinical dimension, encompassing factors such as the Management of pain/suffering and post-operative fragility, to include the compromised autonomy (Vulnerability of the elderly) and the Emotional burden of the disease. This state is exacerbated by patient Polymedication and Memory/cognitive deficits, is perceived as a violation of human dignity, mandating a protective intervention.

“…*the most specific oncology and perioperative care is a vulnerability in extremis… it is, and I think that is what makes the biggest difference, because we know that these are extremely vulnerable and sensitive people with low energy levels, autonomy, and ability to perform their daily activities, take care of themselves, and be comfortable*…”

(H1, Participant Nurse 1, ICU)

“*The post-operative status of these patients always involves a very weak pain control… I don’t think it is very well controlled. It is poorly managed pain*.”

(H1, Participant Nurse 3, ICU)

“…*situations of greater vulnerability, of deficiency, whether emotional or otherwise, it is multidimensional, they are situations of greater fragility, they are situations where we have to be more alert to advocate for that person*.”

(H1, Participant Nurse 4, ICU)

“…*it would not be extreme to say that it is an attack on human dignity, but it places people in a situation of vulnerability and fragility, and we must protect their dignity as much as possible*”

(H1, Participant Nurse 4, ICU)

➢ Facilitating Forces and Constraining Forces

The Facilitating Forces and Constraining Forces configure the intervening conditions, institutional and professional nature that model the nurse’s capacity for agency in the advocacy process. The Family as Anchor emerges as an attenuating and mediating element, serving as the guarantor of the patient’s support and great confidence, functioning as an irreplaceable resource in Family at the forefront of decision-making.

“*Here, the family has a lot of influence*.”

(H1, Participant Nurse 3, ICU)

“*The family holds significant weight here. Visiting hours are very extended, they spend many hours here, and therefore, yes, the family holds significant weight here*.”

(H1, Participant Nurse 3, ICU)

“*The clinical team speaks with the family member first; the family member then enters and stands beside the person, and often information is provided to them, that is, to the patien, and that information is validated with the family member*.”

(H1, Participant Nurse 4, ICU)

“…*it interferes with their family dynamics, which is that some took care of their grandchildren and then no longer*…”

(H1, Participant Nurse 2, ICU)

Barriers such as the Devaluation of the nurse’s opinion, the fact that the Surgeon’s opinion prevails, and Lack of communication constitute Constraining Forces, actively inhibiting ethical action.

“*That was a failure to advocate, because I wanted to advocate, but I couldn’t! I didn’t have it… well, I just didn’t… going against it is very difficult, you know? When I say this, that we nurses do this, I know it’s difficult, attention*…”

(H1, Participant Nurse 1, ICU)

Conversely, Postgraduate specialization and Team support configure themselves as Facilitators of Professional Practice, granting the nurse the necessary authority and expertise to intervene effectively.

“…*When we finish the course, our attention is very focused on technique, and advocating implies having effective management of technique, but also having this entire relational component and specific skills in communication, emotional management, and cognitive management; therefore, I think it is something complex*.”

(H1, Participant Nurse 4, ICU)

### 3.2. Strategies

The Strategies delineate the dynamic actions and interactions undertaken by nurses aimed at mitigating vulnerability and promoting the patient’s best interests, being grouped into two procedural domains:

➢ Advocating for the person’s best interest

This strategy focuses on the proactive defense of the patient’s dignity, safety, and autonomy, grounded in the Knowing and Empowerment dyad. This domain encompasses critical actions such as Advocating for the person’s best interest, Understanding the patient’s wishes, and ensuring Informed decision-making. Actions like knowing the person, establishing greater intimacy with the patients, and listening between the lines are crucial for determining what is best for the patient and knowing when to intervene to maximize protection.

“*I think that today, more than empowerment, I would say that advocacy in current times, they are two married concepts, has even more force! Because today’s patient is not the patient of 20 years ago; I think they are a much more empowered and much more autonomous patient, that is beyond any doubt*.”

(H1, Participant Nurse 1, ICU)

“*To advocate is to defend that person’s best interest, or to try to understand whether that person is informed, whether they can make an informed decision, how they are emotionally, to understand their beliefs, to understand their values—that is, to try to look at that person from a more multidimensional perspective and in all their complexity*.”

(H1, Participant Nurse 1, ICU)

➢ Promoting a Meaningful Understanding of Information

This strategy emphasizes the nurse’s communication role as an interprofessional link (link between classes). The primary action resides in the Deconstructing and Transmitting Information, aiming to simplify technical jargon and validating information with the patient and family. This mediation is vital for the full exercise of self-determination and is directly related to the necessity of information transmission effective between different specialties, including the understanding of drug metabolism and the possibility of drafting advance directives.

“…*you have to read between the lines, there are some patients who completely delegate to their family*!”

(H1, Participant Nurse 2, ICU)

“…*it is much easier to explain what might happen and for them to understand what will happen next, and for them to be much less frightened, and for the family to be much less frightened*.”

(H1, Participant Nurse 3, ICU)

“…*but I felt it was a job well done*.”

(H1, Participant Nurse 2, ICU)

**Figure 2:**
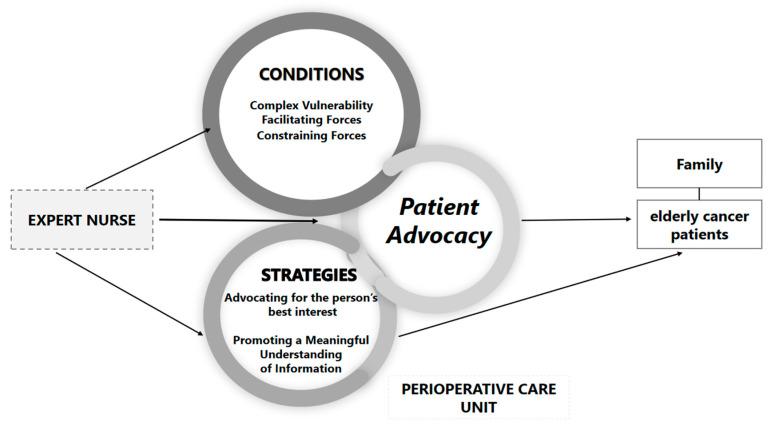
Dynamic Expert Nurse Advocacy Cycle.

The following scheme (Figure 2), illustrates the Conditions and Strategies that potentiate advocacy in this context, culminating in the Dynamic Expert Nurse Advocacy Cycle, the Provisional Core Category. This processual model describes the nurse’s intentional response to the patient’s Complex Vulnerability, being structurally modulated by the Facilitating and Constraining Forces of the institutional context. The nurse’s action is manifested in a Dual Strategic Response, which articulates the relational domain (Advocating) and the communicative domain (Promoting a Meaningful Understanding of Information), culminating in the safeguarding of patient autonomy and dignity.

## 4. Discussion

This study focuses on exploring the conditions and strategies guiding nurse advocacy for elderly oncology patients in perioperative intensive care units. Utilizing a GT approach, this investigation is part of a broader, ongoing study, which also includes patient interview, aimed at constructing and validating a substantive theory regarding this phenomenon.

The critical nature of acute and perioperative environments, where patients are in a state of heightened vulnerability due to surgery and intensive therapy, makes advocacy an indispensable practice. Previous studies emphasise the urgency of implementing nursing advocacy in the perioperative phase, recognising the fundamental role of nurses in communicating with and safeguarding patients with diminished self-determination. At the same time, advocacy promotes more effective communication between healthcare professionals, culminating in increased safety for individuals under care [5,26].

In this context, advocacy emerges as an essential act of respect for the person’s dignity, working to ensure the pain/suffering control of the elderly individual in a state of complex vulnerability. This finding is corroborated by the literature, which highlights the nurse’s role in valuing patient instructions, needs, and how the health condition affects them, emphasizing the physical and psycho-emotional dimensions. This profound appreciation of the human dimension and individual needs underscores the nurse’s protective function in safeguarding patient dignity, particularly within an environment that is, by its nature, depersonalizing and invasive [27].

The family is consistently recognized by participants as fundamental and an anchor, frequently being placed at the forefront of decision-making in patients presenting with memory or cognitive deficits. Consequently, nurses must mediate by informing and clarifying issues to the family, managing expectations, and addressing potential interference in family dynamics. Flexible visiting hours are identified as a key facilitator in this process. The importance of the family as a pillar of support is further underscored in crisis contexts (e.g., the COVID-19 pandemic), where nurse advocacy focuses on valuing the patient’s social and family life, emphasizing the crucial need to maintain connection even within restrictive environments [28].

However, the exercise of this advocacy is structurally constrained, a practice modulated by the Facilitating and Constraining Forces. Participants highlighted the prevalence of organizational barriers, notably the devaluation of the nurse’s opinion and the tendency for the surgeon’s judgment to prevail. Furthermore, the lack of communication and the absence of pre-defined protocols actively hinder advocacy action. Studies conducted in emergency departments and intensive care units confirm that the capacity to advocate is consistently compromised by institutional limitations, resource scarcity, and organizational pressures that restrict the effective defence of patients’ interests [29]. In contrast, postgraduate specialization and advanced skills (emotional and cognitive) emerge as Facilitating Conditions that empower the nurse for advocacy [30], conferring the necessary authority and expertise to mitigate these barriers.

Study participants also highlighted different conceptions of what it means to advocate for the person, including concepts such as advocating for the person’s best interest, understanding the patient’s wishes, empowerment, and informed decision-making. Evidence reiterates that in nursing, advocacy is fundamentally defined as defending the person’s best interest, responding to their needs, and honouring their wishes, a premise widely supported in the literature [6,7,8].

Within this scope, through the analysis of the interviews, codes emerged focusing on central communication strategies, such as deconstructing complex information, validating information, linking between classes, and information transmission. Communication strategies are essential, requiring the nurse to know the person in their entirety, including their beliefs, culture, and values, and to be able to interpret silences and read between the lines to understand their unspoken needs [31]. Additionally, the code “link between classes” was highlighted, showing, as evidence reiterates, that the nurse functions as a link between different professional groups, acting as a crucial communicator who must deconstruct complex information and validate it for the patient and family [31].

Simultaneously, direct action strategies are observed, in which nurses mobilize their in-depth clinical knowledge of drug metabolism and various treatments to maximize patient protection [16]. This direct action includes initiatives such as seeking a second opinion and ensuring that the patient’s wishes prevail in critical situations, such as refusing treatment or drafting advance directives, thereby safeguarding their autonomy and dignity [16,30,32]. Empirically, a correlational study established a significant, positive relationship between nurses’ advocacy behaviours and their professional values [33]. This evidence directly supports our conclusion that advocacy transcends a mere prescribed role; it is an ethical and deeply rooted manifestation of core professional nursing values.

The dynamic expert nurse advocacy cycle emerges as the provisional core category, offering a processual model that explains how the ethical role of advocacy is activated and operationalized within high-complexity settings. This model demonstrates that advocacy is an intentional response to the patient’s complex vulnerability, being structurally modulated by the facilitating and constraining forces of the institutional context. By moving beyond descriptive ethical frameworks, this cycle systematically articulates the translation of clinical experience into actionable strategy, providing a valuable conceptual framework that can guide policy planning and the design of future large-scale quantitative studies aimed at testing the relationships proposed herein.

A recent article emphasises that nurses act as ethical stewards in the dynamic healthcare landscape, and their duty to advocate is inseparable from other responsibilities, such as whistleblowing on patient safety concerns [34]. This perspective reinforces that advocacy is a moral duty, not an optional action. This duty takes on special importance when we consider that preserving patient dignity is a fundamental human right, the fragility of which is exacerbated in the ICU, as demonstrated by a qualitative study [35]. This study revealed that dignity is maintained through active interventions of person-centred care, respect for autonomy, and empathic communication, all of which are central elements of advocacy. Thus, our findings are in line with international understanding, positioning advocacy as the primary ethical mechanism that nurses use to protect patients from loss of dignity and ensure their safety in highly vulnerable environments.

## 5. Strengths and Limitations

The strengths of this study essentially lie in its methodological approach and strategic positioning within the research context. The rigorous use of Grounded Theory, whose application allows for the generation of fragments of a substantive theory about the phenomenon of advocacy for older adults in the perioperative context, stands out. Although this model is circumscribed to the specific context, it established the essential conceptual categories regarding the conditions and strategies of the phenomenon under study, which become the theoretical foundation for the development of Formal Theory in subsequent phases of the project. Additionally, the fact that the study was conducted in an International Reference Oncology Unit provides great credibility and depth to the data, ensuring that the insights on managing ethical dilemmas and institutional barriers were collected in a setting of excellence and maximum clinical complexity. This focus on a highly critical population (Older adults, Cancer, ICU) fills a knowledge gap and holds transferability potential under similar contextual conditions.

Nevertheless, the limitations of this study are inherent to the nature of qualitative research and to its execution stage. The main methodological limitation to be recognised is the limited transferability of the findings. Although the small sample size (n = 6) was sufficient to achieve the theoretical saturation required by Grounded Theory, the focus on a single context, a Perioperative Intensive Care Unit in a Reference Oncology Institution, means that the results are intrinsically shaped by its specific cultural environment and resources. The applicability of the theory must therefore be interpreted through conceptual analogy, with the recognition that the identified Barriers and Facilitators may be deeply rooted in the organisational culture of the study. However, it is crucial to emphasise that the purpose of this work is not generalisation, but rather the creation and validation of the still-developing theory on this phenomenon.

## 6. Conclusions

This investigation, in its provisional stage of Grounded Theory, systematically articulated the Conditions and Strategies that guide expert nurse advocacy for elderly oncology patients in the perioperative ICU. The study confirms that the practice is an imperative response to the patient’s Complex Vulnerability, and is structurally mediated by organizational barriers and facilitators. The Dynamic Expert Nurse Advocacy Cycle emerges as a processual model that translates experience into strategic practice, highlighting the necessity of a dual, relational and informational approach for safeguarding autonomy and dignity. The findings underscore the urgency of public health policies that recognize and reinforce the nurse’s role as an *ethical steward*, providing a theoretical and practical framework to guide future professional development, necessary institutional support, and advanced training for *advanced nursing*.

## Figures and Tables

**Table 1:** Demographic data of clinical nurses.

Gender	Clinical Nurses (*n* = 6)
Male Female	2 4
Age	
25–30 31–40 41–50	0 2 4
Years of Professional Experience	
1–10 11–20 21–30	0 3 3
Professional Experience in the Field	
6–10 11–22	5 1
Postgraduate Education	
Master	3
Nursing Specialization	4

**Table 2:** Examples of the Evolution of Coding: From Open to Selective (Grounded Theory).

	**CONDITIONS**	
**Open Coding**	**Axial Coding**	**Selective Coding**
Vulnerability of the elderly Emotional burden of the disease Violation of human dignity Management of pain/suffering Polymedicated patients Memory/cognitive deficits Family support Family at the forefront of decision-making Flexible visiting hours Devaluation of the nurse’s opinion Surgeon’s opinion prevails Lack of communication Postgraduate specialization Team support	Physical, Emotional, and Cognitive Frailty Family as Anchor Family at the forefront Barriers of Professional Practice Facilitators of Professional Practice	Complex Vulnerability Facilitating Forces and Constraining Forces
	**STRATEGIES**	
**Open Coding**	**Axial Coding**	**Selective Coding**
Advocating for the person’s best interest Understanding the patient’s wishes Empowerment Informed decision-making Deconstructing complex information validating information link between classes information transmission maximizing protection drafting advance directives understanding drug metabolism	Knowing and Empowerment Deconstructing and Transmitting Information	**Advocating for the person’s** **best interest** **Promoting a Meaningful Understanding of Information**

## Data Availability

The data is available upon request, but is not publicly and unconditionally accessible because this restriction is an ethical and legal requirement defined in our research protocol.

## Abbreviations

The following abbreviations are used in this manuscript:

GT	Grounded Theory
ICU	Intensive Care Unit
